# Algicidal bacterium CZBC1 inhibits the growth of *Oscillatoria chlorina*, *Oscillatoria tenuis*, and *Oscillatoria planctonica*

**DOI:** 10.1186/s13568-019-0872-8

**Published:** 2019-09-11

**Authors:** Xiao-Juan Hu, Yu Xu, Hao-Chang Su, Wu-Jie Xu, Li-Hua Wang, Yun-Na Xu, Zhuo-Jia Li, Yu-Cheng Cao, Guo-Liang Wen

**Affiliations:** 1 Key Laboratory of South China Sea Fishery Resources Exploitation & Utilization, Ministry of Agriculture; Key Laboratory of Fishery Ecology and Environment, Guangdong Province; South China Sea Fisheries Research Institute, Chinese Academy of Fishery Sciences, Guangzhou, 510300 China; 2 Shenzhen Base South China Sea Fisheries Research Institute, Chinese Academy of Fishery Sciences, Shenzhen, 518121 China

**Keywords:** Algicidal bacteria, *Oscillatoria* spp., Algicidal effect, Effective concentrations

## Abstract

Frequent harmful cyanobacteria blooms limit the sustainable development of aquaculture. Algicidal bacteria can efficiently control harmful algae without secondary pollution. The algicidal bacteria CZBC1 can lyse *Oscillatoria* spp. and other harmful cyanobacteria, but its effector mechanism and algicidal threshold are unknown. In this study, we examined the algicidal effect of CZBC1 on *O. chlorina, O. tenuis*, and *O. planctonica* by microscopic enumeration and scanning electron microscopy observation. Then, we examined the alginolytic effects of CZBC1 (concentrations 10^3^–10^6^ colony forming units (cfu)/mL) on these three species (concentrations 10^3^–10^6^ cells/mL) to determine the effective concentrations of CZBC1 for *Oscillatoria* spp. alginolysis. Results showed that CZBC1 can directly lyse *O. chlorina* and *O. tenuis* but indirectly lyse *O. planctonica*. When the initial concentration of CZBC1 was 10^6^ cfu/mL, alginolytic effects were high for all three species at all concentrations, and the alginolytic rate could reach 100% in 3–9 days. When the initial concentration of CZBC1 was lower (10^3^ cfu/mL), its inhibitory effects were delayed by 2–5 days, but the cell counts were significantly decreased compared with the control, evidencing significant alginolysis. In addition, the higher the concentration of the algicidal bacteria suspension, the more significant the alginolytic effects. Our results indicate that CZBC1 has different alginolytic mechanisms for *O. chlorina*, *O. tenuis*, and *O. planctonica*, and that different initial concentrations of CZBC1 have different alginolytic effects on these algal species.

## Introduction

Eutrophication of water bodies, frequent cyanobacteria blooms, and other environmental problems have not only greatly affected the natural environment but also severely hindered the sustainable development of aquaculture (Cao et al. 2014b; Sun et al. 2017). *Oscillatoria* species are dominant cyanobacteria in prawn aquaculture ponds (Liu et al. 2011), and their abundance in these systems has been significantly and negatively correlated with prawn yield (Cao et al. 2014b). How to effectively control harmful cyanobacteria, particularly *Oscillatoria* sp., blooms has become a key area of concern in aquaculture research (Li et al. 2016). Studies have shown that algicidal bacteria can lyse algal cells, either through direct contact or through the release of specific or non-specific extracellular products (Yang et al. 2013; Chen et al. 2015). They can also compete for nitrogen and phosphate salts to inhibit the growth of microalgae, and the use of algicidal bacteria for controlling harmful algae is highly efficient and lacks secondary pollution (Xi et al. 2016). However, most studies on algicidal bacteria have focused on natural seas, lakes, rivers, or other open water bodies (Han et al. 2001; Miao et al. 2002), and the species investigated have mostly been from the same aquatic environments or from the phycosphere (Jin et al. 2010; Yang et al. 2013). Differences between open water bodies and aquaculture ponds may lead to unexpected ecological or biosafety problems if the results from the aforementioned studies are directly applied to aquaculture ponds. Therefore, conducting research on the application of algicidal bacteria in aquaculture ponds will contribute to solve the problem of harmful cyanobacteria blooms in aquaculture ponds (Wang et al. 2012).

The alginolytic mechanisms of algicidal bacteria usually involve both direct and indirect alginolysis. The former refers to algicidal bacteria coming into direct contact and actively attacking algal cells or even invading algal cells to destroy their cell structure and lysate microalgae. The latter refers to indirect attacks on the host, which mainly involve competing for limiting nutrients, forming biofilms, or releasing specific or non-specific extracellular substances for alginolysis (Guan et al. 2014; Li et al. 2015). Algicidal bacteria attack algae by different mechanisms, with some bacterial strains exhibiting distinct mechanisms for different algal species. Furthermore, algicidal bacteria require specific concentrations to carry out alginolytic functions, and the concentration threshold differs among bacterial strains. Research on alginolytic bacteria aims to delineate these different mechanisms and concentrations. Manage et al. (2000) found that an initial concentration of 10^3^ colony forming units (cfu)/mL of the bacterium *Alcaligenes denitrificans* could lyse *Microcystis aeruginosa*. Peng et al. (2003) reported the lower limits for alginolytic concentrations of *Staphylococcus* sp. M5, *Bacillus* sp. M8, and *Arthrobacter* sp. M13 as 10^5^ cfu/mL, 10^6^ cfu/mL, and 10^5^ cfu/mL, respectively. Pei et al. (2005) found that the greater the initial concentration of the algicidal bacterium P07, the more significant the alginolytic effect. When bacterial concentration was 2.4 × 10^7^ cfu/mL, 81.67% of *M. aeruginosa* was removed after 3 days. Thus, the initial concentrations of algicidal bacteria determine alginolytic results, and determining the most effective initial concentration is important for the industrial application of algicidal bacteria.

Previously, our team obtained the algicidal *Bacillus cereus* CZBC1 strain from screening aquaculture ponds, and found that this strain could effectively control the growth of harmful algae (Wang et al. 2016), thereby having potential application in aquaculture (Xu et al. 2019). In the present study, we tested the alginolytic modes of CZBC1 for the common aquaculture cyanobacteria *Oscillatoria chlorina, Oscillatoria tenuis*, and *Oscillatoria planctonica*. We analyzed the effects of different initial concentrations of CZBC1 on the growth of different initial concentrations of algal cells and examined the potential effector mechanisms of CZBC1 on these species. Our findings provide a theoretical basis for the industrial application of CZBC1.

## Materials and methods

### Test samples

Strain CZBC1 was provided by the South China Sea Fisheries Research Institute, Chinese Academy of Fishery Sciences, and it was identified as *Bacillus cereus*. This strain has alginolytic effects on *Oscillatoria* sp., *Microcystis* sp., and other cyanobacteria. The strain was stored at the China Center for Type Culture Collection (CCTCC no.: M2013130), and a national invention patent was issued (ZL201310203745.3; Cao et al. 2014a).

We selected *O. chlorina*, *O. tenuis*, and *O. planctonica* for testing the alginolytic effects of CZBC1. *Oscillatoria chlorina* was obtained from the algal preservation laboratory of South China Sea Fisheries Research Institute, Chinese Academy of Fishery Sciences, and *O. tenuis* (FACHB-1052) and *O. planctonica* (FACHB-708) were obtained from the freshwater algal species repository of the Institute of Hydrobiology, Chinese Academy of Sciences.

### Alginolytic effects of CZBC1 against the three *Oscillatoria* species

#### Experimental design and enumeration of algae

A small amount of CZBC1 stored in preserving solution was inoculated to a nutrient agar plate under aseptic conditions and then activated at 28 °C for 24 h. A few activated CZBC1 colonies were inoculated into a 250-mL sterile conical flask containing 100 mL of sterile nutrient broth, and the flask was placed in a shaking incubator at 30 °C for 16 h. The CZBC1 bacterial suspension was collected and centrifuged at 9391×*g* for 2 min. The resulting supernatant was separated from the pellet and used as a sterile filtrate, while the pellet was washed with sterile water and centrifuged again at 9391×*g* for 2 min; the resulting supernatant was then discarded. This process was repeated three times. The collected pellet was resuspended in sterile water to the required concentration of bacterial suspension for subsequent experiments.

*Oscillatoria chlorina*, *O. tenuis*, or *O. planctonica* was inoculated aseptically into a sterile 1000-mL conical flask containing 600 mL of sterile BG11 culture broth and placed in an illuminated incubator. Expansion culture was carried out under 28 °C, 2500 lx light intensity, and a light/dark cycle of 12/12 h. The culture flasks were periodically shaken throughout the day to prevent the algal cells from attaching to the walls, and the culture was carried out until the algae reached the logarithmic growth phase.

Next, the CZBC1 suspension or the sterile filtrate (10 mL) was added to a sterilized 250-mL conical flask containing 90 mL of *O. chlorina*, *O. tenuis*, or *O. planctonica* in the logarithmic growth phase. The volume of the co-culture system was 100 mL. The initial concentration of *O. chlorina*, *O. tenuis*, and *O. planctonica* was 6.8 × 10^6^ cells/mL, 1.21 × 10^7^ cells/mL, and 3.87 × 10^6^ cells/mL, respectively. In the culture system, the concentration of CZBC1 was 10^7^ cfu/mL and an equal volume of sterile water was added to the control group. Parallel triplicates were set up for each group and placed in an illuminated incubator. The flasks were cultured at 28 °C, 2000 lx light intensity, and a light/dark cycle of 12/12 h for 7 days. After the bacterial suspension and sterile filtrate were added to the algal solution, sampling was carried out immediately and then once every 24 h. The samples were fixed with formaldehyde and optical microscopy was used to observe algal cell morphology and to enumerate algal cells.

#### Scanning electron microscopy observation of algal cell morphology

After the CZBC1 suspension or the sterile filtrate was added to the algal solution, sampling was carried out immediately and then once every 6 h. The volume of each sample was 5 mL. The samples were centrifuged at 438×*g* for 10 min to collect algal cells, which were fixed with 2.5% glutaraldehyde for more than 3 h. The algal cells collected by centrifugation were washed 4–6 times with 0.1 M phosphate buffer for 20 min/wash. Then, ethanol dehydration was performed using 30%, 50%, 70%, and 90% for 15 min each, followed by three anhydrous ethanol dehydration steps for 15 min each time. The cells were placed in tert-butanol three times, 15 min each time, and, after collecting algal cells, tert-butanol was added, and the cells were added to a slide for carbon dioxide critical point drying, metal spraying, and observation by scanning electron microscopy.

### Effects of different initial concentrations of CZBC1 on the three *Oscillatoria* species growth

The initial concentrations of CZBC1 used were 10^3^ cfu/mL (J-3 group), 10^4^ cfu/mL (J-4 group), 10^5^ cfu/mL (J-5 group), and 10^6^ cfu/mL (J-6 group). An equal volume of sterile water was added to the control group. Parallel triplicates were set up for each group (Control, J-3, J-4, J-5 and J-6 group). In the culture system, the concentrations of *O. chlorina*, *O. tenuis*, and *O. planctonica* were 10^3^ cells/mL, 10^4^ cells/mL, 10^5^ cells/mL, and 10^6^ cells/mL. Different concentrations of CZBC1 suspension (10 mL) were collected and added to 90 mL of algal solution at different concentrations. The flasks were cultured at 28 °C, 2000 lx light intensity, and a light/dark cycle of 12/12 h for 9 days. Sampling was carried out immediately after adding the bacterial suspension, and then once every 24 h.

### Data analysis

SPSS 20.0 software (IBM) was used to carry out one-way analysis of variance (ANOVA) followed by Duncan’s test. The significance level was set as *P *< 0.05. Alginolysis rate (%) was calculated as (1 − N_t_/N_0_) × 100, where N_t_ is the algal cell count at time t, and N_0_ is the initial algal cell count (Lin et al. 2014). Algal cell count reduction rate (%) was calculated as (1 − N_Jt_/N_Ct_) × 100, where N_J_t is the algal cell count at time t in the group to which the bacterial suspension was added and N_Ct_ is the algal cell count at time t in the control group.

## Results

### Alginolytic effects of CZBC1 against the three *Oscillatoria* species

#### Alginolytic effects of CZBC1 against *O. chlorina* and *O. tenuis*

**Fig. 1:**
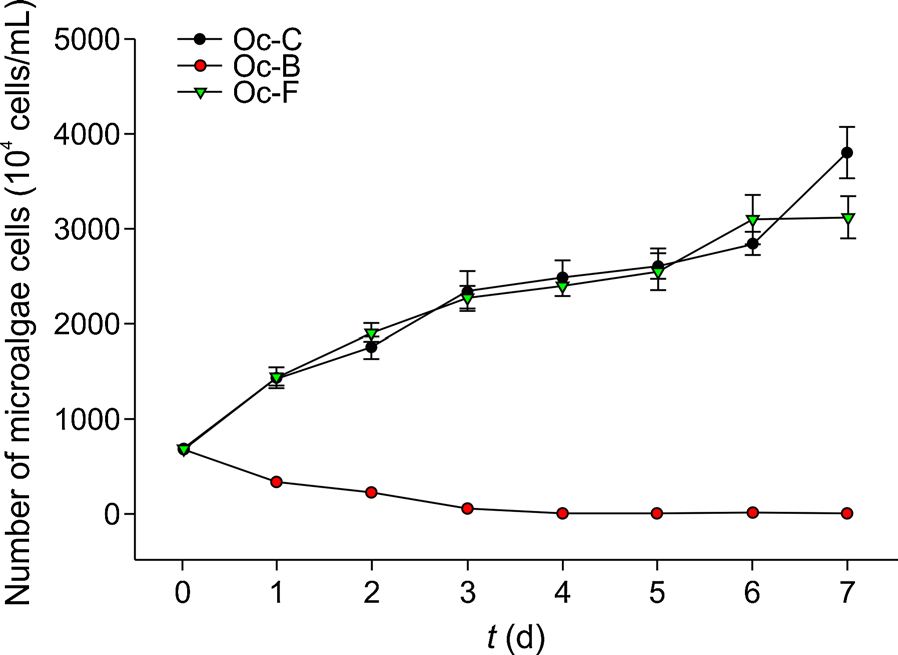
Effects of CZBC1 on *Oscillatoria chlorina* growth. Oc-C: *O. chlorina* control group; Oc-B: *O. chlorina* + bacterial suspension group; Oc-F: *O. chlorina* + sterile filtrate group

**Fig. 2:**
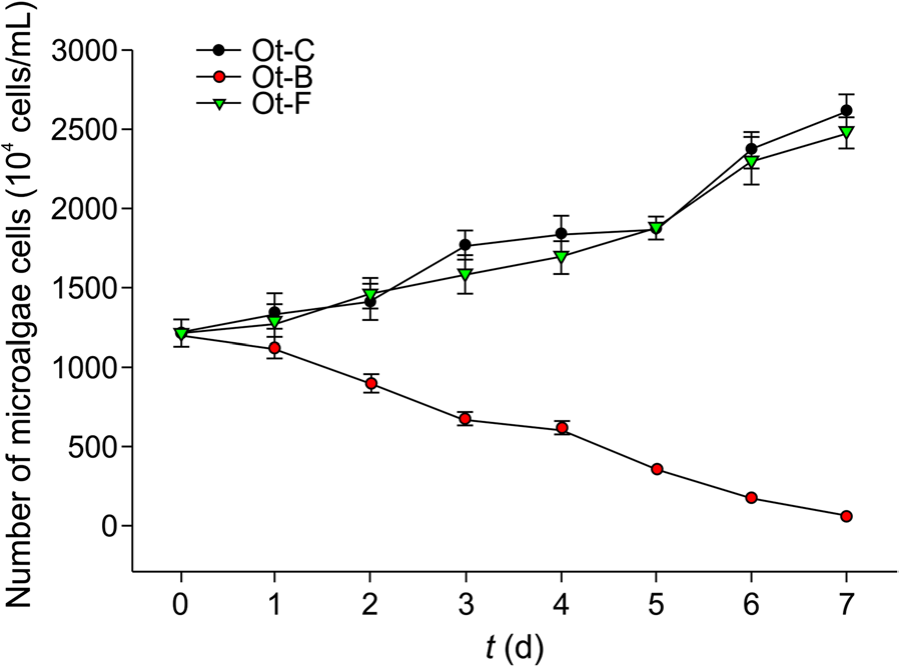
Effects of CZBC1 on *Oscillatoria tenuis* growth. Ot-C: *O. tenuis* control group; Ot-B: *O. tenuis* + bacterial suspension group; Ot-F: *O. tenuis* + sterile filtrate group

*Oscillatoria chlorina* and *O. tenuis* showed the same trend. In the control groups, *O. chlorina* (Oc-C) and *O. tenuis* (Ot-C) showed logarithmic growth, and the algal cell counts on day 7 were 3.79 × 10^7^ cells/mL and 2.62 × 10^7^ cells/mL, respectively. The cell counts of both species started to decrease in the bacterial suspension groups (Oc-B and Ot-B), where *O. chlorina* cells died by day 4 and *O. tenuis* cell count on day 7 was 5 × 10^5^ cells/mL. Microscopy analysis showed alginolysis of *O. chlorina* and *O. tenuis* cells, and flocculation could be observed at the bottom of the conical flasks. In the sterile filtrate groups (Oc-F and Ot-F), the cells increased to 3.11 × 10^7^ cells/mL and 2.48 × 10^7^ cells/mL on day 7, which was not significantly different from that observed for the control group (Figs. 1, 2). These results suggested that CZBC1 can lyse *O. chlorina* and *O. tenuis*, and that its extracellular products do not affect their growth.

**Fig. 3:**
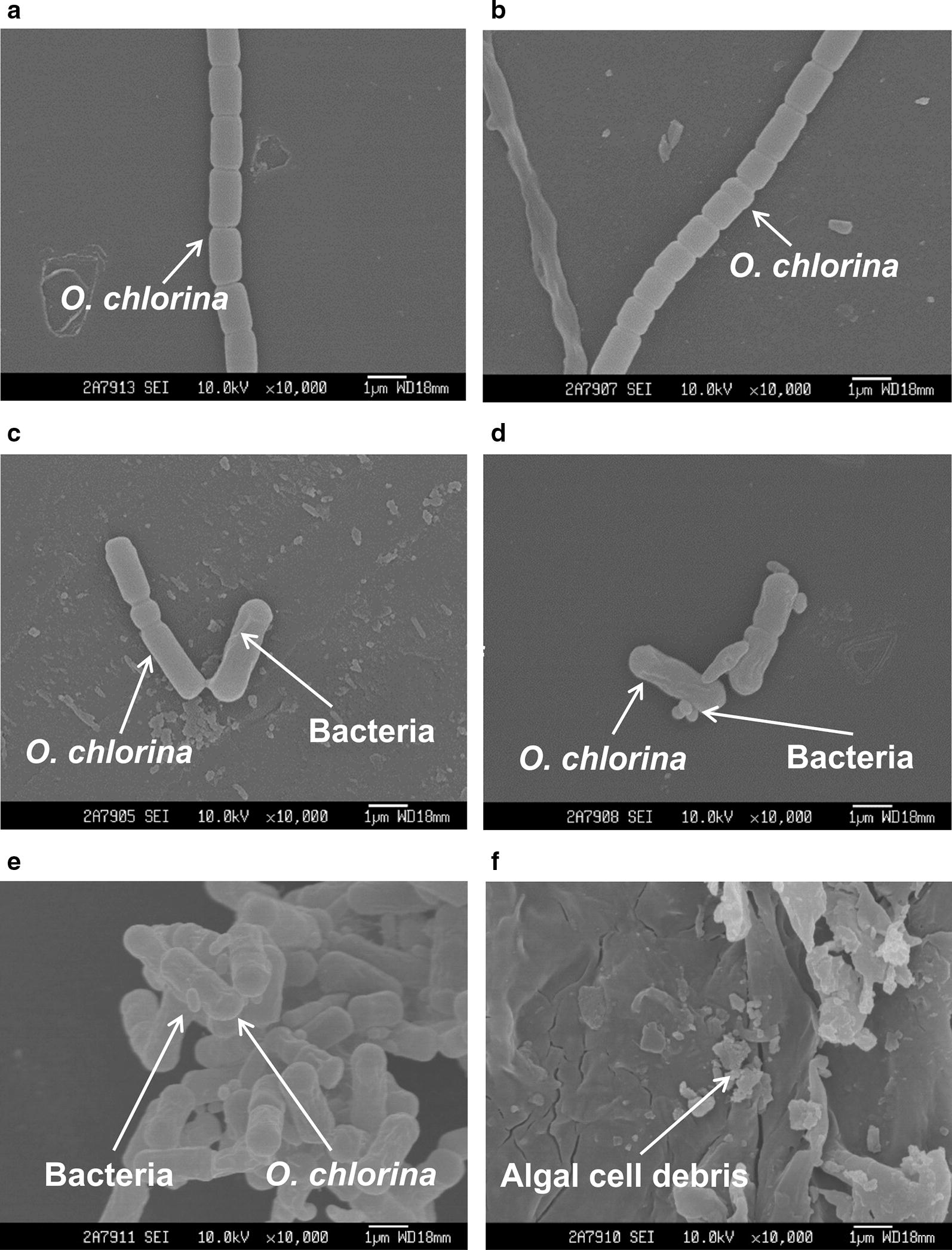
Scanning electron micrographs of the effects of CZBC1 on *Oscillatoria chlorina* cells. **a** initial stage in Oc-C and Oc-F; **b** initial stage in Oc-B; **c** early stage of alginolysis in Oc-B; **d**, **e** middle stage of alginolysis in Oc-B; **f** late stage of alginolysis in Oc-B. Oc-C: *O. chlorina* control group; Oc-B: *O. chlorina* + bacterial suspension group; Oc-F: *O. chlorina* + sterile filtrate group

**Fig. 4:**
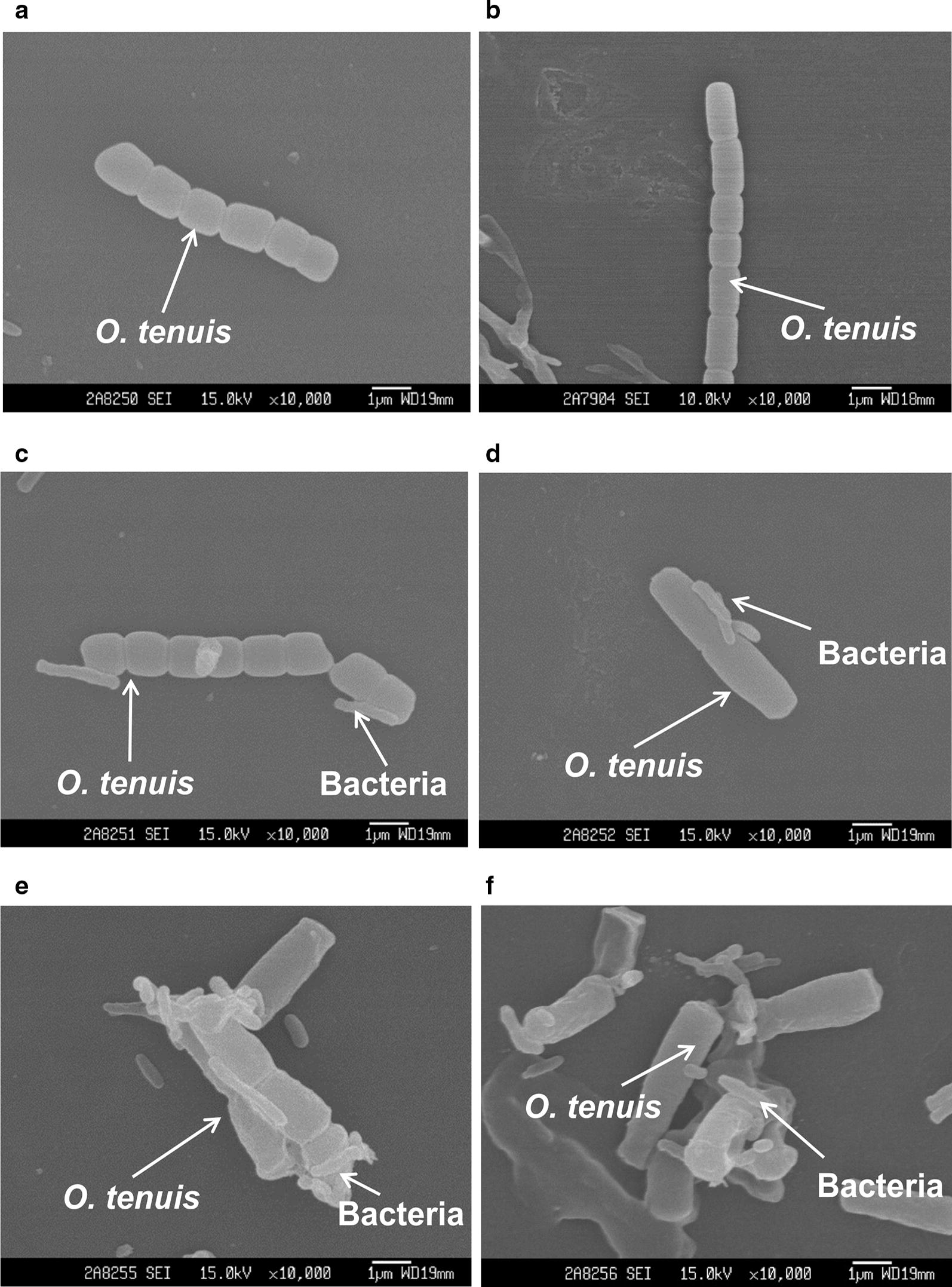
Scanning electron micrographs of the effects of CZBC1 on *Oscillatoria tenuis* cells. **a** Initial stage in Ot-C and Ot-F. **b** Initial stage in Ot-B. **c** Early stage of alginolysis in Ot-B. **d** Middle stage of alginolysis in Ot-B. **e**, **f** Late stage of alginolysis in Ot-B. Ot-C: *O. tenuis* control group; Ot-B: *O. tenuis* + bacterial suspension group; Ot-F: *O. tenuis* + sterile filtrate group

Figures 3 and 4 display scanning electron micrographs of the effects of CZBC1 on *O. chlorina* and *O. tenuis* cell morphology. Algal cells in the control and sterile filtrate groups exhibited normal morphology; i.e., multiple algal cells were joined together, the outer filament walls were smooth, and there was no bacterial attachment to the filament surface. In the bacterial suspension group, algal cell morphology was initially identical to that of the control group. During early alginolysis, the filaments started to break, bacterial attachment was observed at the cell–cell junctions, and algal cells started to deform. In the middle stage of alginolysis, algal cells became severely deformed, bacteria were attached to surfaces, and filaments broke up into single cells that clumped together. During later alginolysis, algal cells were lysed into debris, or no intact algal cells were observed.

#### Alginolytic effects of CZBC1 against *O. planctonica*

**Fig. 5:**
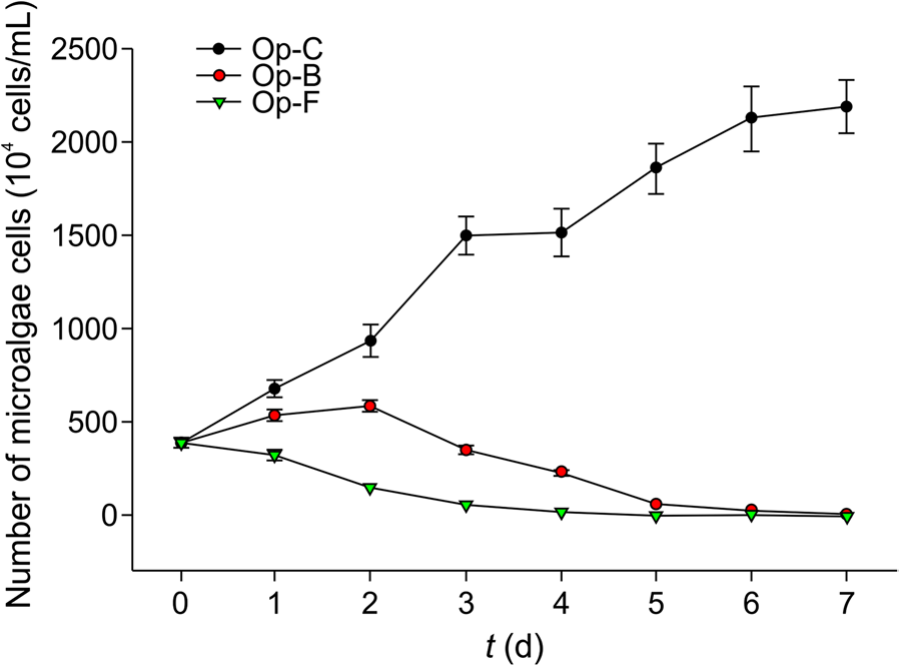
Effects of CZBC1 on *Oscillatoria planctonica* growth. Op-C: *O. planctonica* + control group; Op-B: *O. planctonica* + bacterial suspension group; Op-F: *O. planctonica* + sterile filtrate group

*Oscillatoria planctonica* in the control group (Op-C) showed logarithmic growth, and its cell count on day 7 was 2.19 × 10^7^ cells/mL. In the bacterial suspension group (Op-B), *O. planctonica* cell count increased in the first 2 days, reaching 5.83 × 10^6^ cells/mL on day 2. Subsequently, algal cell count decreased, and all cells died by day 7. In the sterile filtrate group (Op-F), *O. planctonica* started to decrease when the sterile filtrate was added, and all algal cells died by day 5 (Fig. 5). Results showed that the extracellular products of CZBC1 had alginolytic effects on *O. planctonica*. In the bacterial suspension group, *O. planctonica* counts started to decrease after day 2 until all cells were dead. Scanning electron micrographs showed that no bacteria were attached to the cell surfaces. The reason for this may be that, as the experiment progressed, CZBC1 started to proliferate and secrete extracellular products that resulted in alginolysis.

**Fig. 6:**
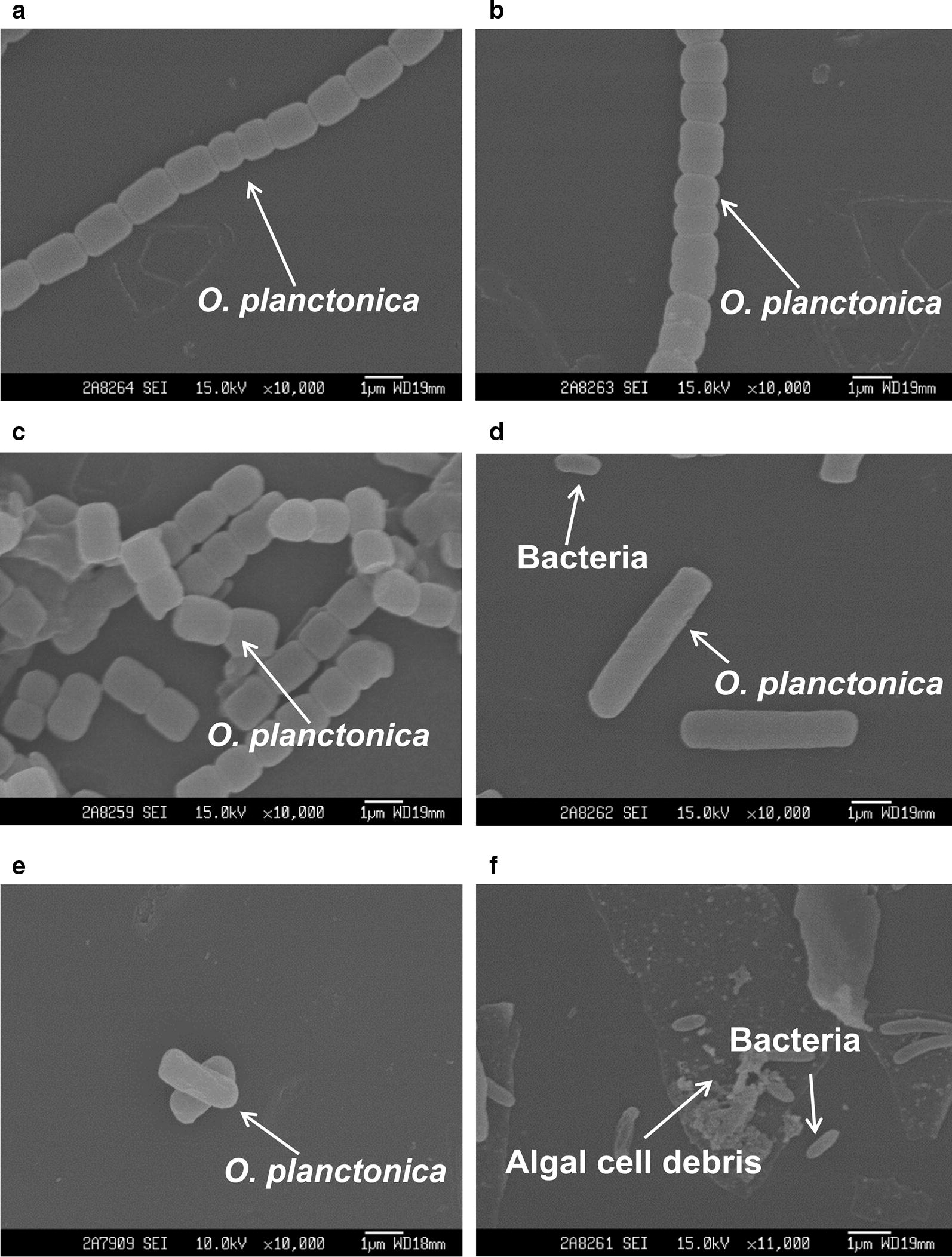
Scanning electron micrographs of the effects of CZBC1 on *Oscillatoria planctonica* cells. **a** Initial stage in Op-C. **b** Initial stage in Ot-B and Op-F. **c** Early stage of alginolysis in Ot-B and Op-F. **d**, **e** Middle stage of alginolysis in Ot-B and Op-F. **f** Late stage of alginolysis in Ot-B and Op-F. Op-C: *O. planctonica* + control group; Op-B: *O. planctonica* + bacterial suspension group; Op-F: *O. planctonica* + sterile filtrate group

The effect of CZBC1 on *O. planctonica* cell morphology differed from that on *O. chlorina* and *O. tenuis* (Fig. 6). In Op-C, multiple algal cells joined to form filaments and no bacteria were attached at the filament surface. In Op-B and Op-F, algal cell morphology was initially identical to that of Op-C. During early alginolysis, the filaments started to break, and no bacterial attachment was observed at the algal cell surface. In the middle stage of alginolysis, the filaments completely broke into single cells, algal cells became severely deformed, and no bacteria were attached to algal cell surfaces. During later alginolysis, algal cell debris could be seen, and no intact algal cells were observed.

### Effects of different initial concentrations of CZBC1 on the alginolysis of the three *Oscillatoria* species

#### Effects of different initial concentrations of CZBC1 on *O. chlorina* alginolysis

**Fig. 7:**
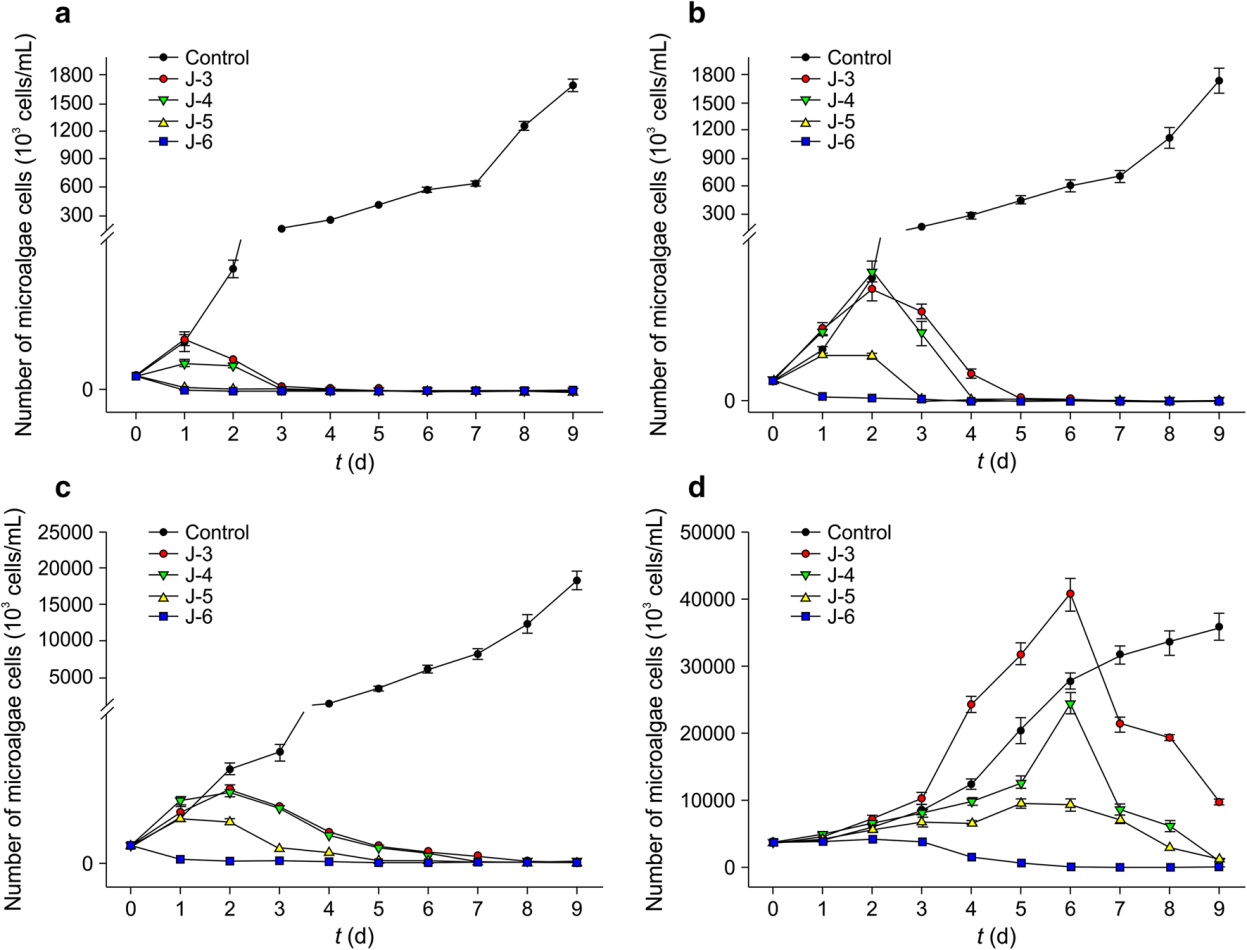
Alginolytic effects of different initial concentrations of CZBC1 on *Oscillatoria chlorina* at different initial concentrations. **a** 10^3^ cells/mL. **b** 10^4^ cells/mL. **c** 10^5^ cells/mL. **d** 10^6^ cells/mL

When the initial cell count of *O. chlorina* was 10^3^ cells/mL (Fig. 7a), cells in the control group exhibited logarithmic growth and cell count was 5.6 × 10^4^ cells/mL on day 2. This increased to 1.68 × 10^6^ cells/mL on day 9, which was significantly higher than that of the bacterial suspension group (*P* < 0.05). In the J-3 and J-4 groups, the cell count increased in the first 2 days reaching 1.47 × 10^4^ cells/mL and 1.27 × 10^4^ cells/mL, respectively. Subsequently, the cell count decreased. On day 3, the alginolysis rate was 76.39% and 84.72%, respectively, and by day 6 all *O. chlorina* cells of both groups had died. In the J-5 and J-6 groups, the cells started to decrease after the bacterial suspension was added. On day 1, the alginolysis rate was 75.69% and 86.11%, respectively, and all *O. chlorina* algal cells had died by day 5 and day 4, respectively.

When the initial cell count of *O. chlorina* was 10^4^ cells/mL (Fig. 7b), cells in the control group gradually increased as culture duration increased and cell count was 1.74 × 10^6^ cells/mL on day 9, which was significantly higher than that of the bacterial suspension group (*P* < 0.05). The cell count increased in the J-3, J-4, and J-5 groups after the bacterial suspension was added, reaching a maximum of 5.46 × 10^4^ cells/mL, 6.33 × 10^4^ cells/mL, and 2.3 × 10^4^ cells/mL, respectively. Subsequently, the cell count of the three groups started to decrease, and all algal cells had died by days 7, 7, and 5, respectively. In the J-6 group, the alginolysis rate on day 1 was 78.30% and all algal cells had died by day 5.

When the initial cell count of *O. chlorina* was 10^5^ cells/mL (Fig. 7c), cells in the control group gradually increased as culture duration increased and cell count was 1.83 × 10^7^ cells/mL on day 9, which was significantly higher than that of the bacterial suspension group (*P *< 0.05). The cell count increased in the J-3, J-4, and J-5 groups after the bacterial suspension was added, reaching a maximum of 5.83 × 10^5^ cells/mL, 5.54 × 10^5^, and 3.53 × 10^5^ cells/mL, respectively. Subsequently, the cell count of the three groups started to decrease and all algal cells had died by days 9, 9, and 8, respectively. In the J-6 group, the alginolysis rate on day 1 was 79.55% and all algal cells had died by day 6.

When the initial cell count of *O. chlorina* was 10^6^ cells/mL (Fig. 7d), cells in the control group gradually increased as culture duration increased and cell count was 3.57 × 10^7^ cells/mL on day 9. The cell count first increased before decreasing in the J-3, J-4, and J-5 groups, after the bacterial suspension was added. During the first 6 days, *O. chlorina* growth was faster in the J-3 group than in the control group, and count was 4.05 × 10^7^ cells/mL on day 6, which was higher than that of the control group (2.77 × 10^7^ cells/mL). On day 6, cell count was 2.43 × 10^7^ cells/mL in the J-4 group. The cell counts of the J-3 and J-4 groups significantly decreased, and on day 9 of the experiment, the algal cell count reduction rates of the J-3, J-4, and J-5 groups were 72.73%, 97.02%, and 96.76%, respectively. The alginolysis rate in the J-6 group on day 5 was 84.59%, and all algal cells had died by day 9.

#### Effects of different initial concentrations of CZBC1 on *O. tenuis* alginolysis

**Fig. 8:**
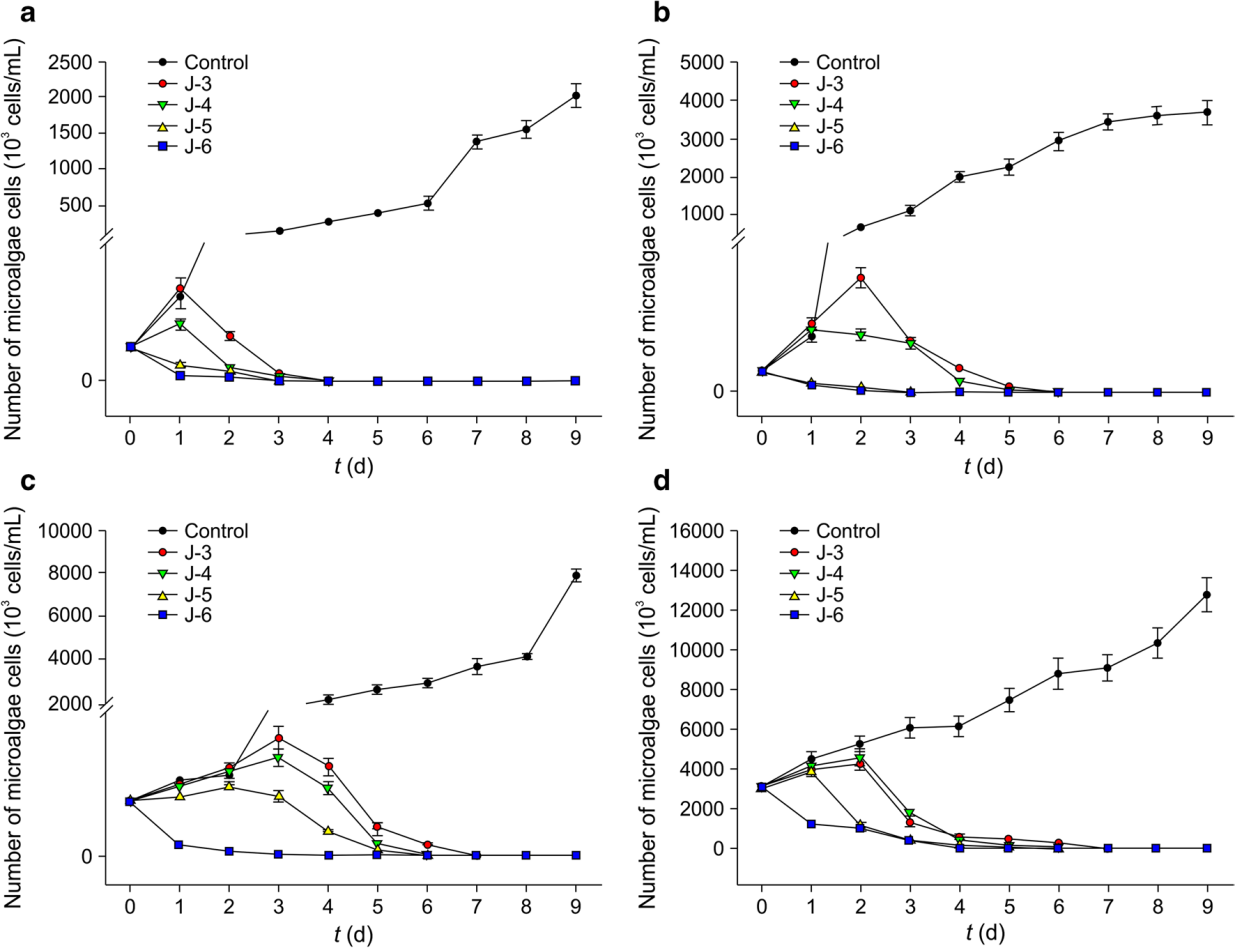
Alginolytic effects of different initial concentrations of CZBC1 on *Oscillatoria tenuis* at different initial concentrations. **a** 10^3^ cells/mL. **b** 10^4^ cells/mL. **c** 10^5^ cells/mL. **d** 10^6^ cells/mL

When the initial cell count of *O. tenuis* was 10^3^ cells/mL (Fig. 8a), cells in the control group exhibited logarithmic growth and cell count was 2.02 × 10^6^ cells/mL on day 9, which was significantly higher than that of the bacterial suspension group (*P* < 0.05). In the J-3 and J-4 groups, the cell count increased on the first day and reached 2.67 × 10^4^ cells/mL and 1.66 × 10^4^ cells/mL, respectively, before decreasing. On day 3, the alginolysis rate for *O. tenuis* was 76.39% and 84.06% in the J-3 and J-4 groups, respectively. By day 4, all *O. tenuis* in both groups had died. In the J-5 and J-6 groups, the cell count started to decrease after the bacterial suspension was added, and all algal cells from these two groups had died by day 3.

When the initial cell count of *O. tenuis* was 10^4^ cells/mL (Fig. 8b), cells in the control group gradually increased as culture duration increased and cell count was 3.67 × 10^6^ cells/mL on day 9, which was significantly higher than that of the bacterial suspension group (*P* < 0.05). In the J-3 and J-4 groups, the cell count increased after the bacterial suspension was added, and the maximum cell count was 2.29 × 10^5^ cells/mL and 1.25 × 10^5^ cells/mL, respectively. Subsequently, algal cell counts in these two groups decreased, and all algal cells had died by day 6. In the J-5 and J-6 groups, the cell count started to decrease after the bacterial suspension was added, and all algal cells from these two groups had died by day 3.

When the initial cell count of *O. tenuis* was 10^5^ cells/mL (Fig. 8c), cells in the control group gradually increased as culture duration increased and cell count was 7.87 × 10^6^ cells/mL on day 9, which was significantly higher than that of the bacterial suspension group (*P* < 0.05). The cell count increased in the J-3, J-4, and J-5 groups after the bacterial suspension was added, reaching a maximum of 1.23 × 10^6^ cells/mL, 1.03 × 10^6^ cells/mL, and 7.15 × 10^5^ cells/mL, respectively. Subsequently, the *O. tenuis* cell count of the three groups started to decrease and all algal cells had died by days 8, 7, and 7, respectively. In the J-6 group, algal cell count started to decrease after the bacterial suspension was added. The alginolysis rate on day 3 was 98.02%, and all algal cells had died by day 4.

When the initial cell count of *O. tenuis* was 10^6^ cells/mL (Fig. 8d), cells in the control group gradually increased as culture duration increased and cell count was 1.27 × 10^7^ cells/mL on day 9. The cell count first increased before decreasing in the J-3, J-4, and J-5 groups, after the bacterial suspension was added, reaching a maximum of 4.20 × 10^6^ cells/mL, 4.54 × 10^6^ cells/mL, and 3.85 × 10^6^ cells/mL, respectively. Subsequently, the cell count of the three groups started to decrease, and all algal cells had died by day 8. In the J-6 group, algal cell count started to decrease after the bacterial suspension was added. The alginolysis rate on day 4 was 98.02%, and all algal cells had died by day 5.

#### Effects of different initial concentrations of CZBC1 on *O. planctonica* alginolysis

**Fig. 9:**
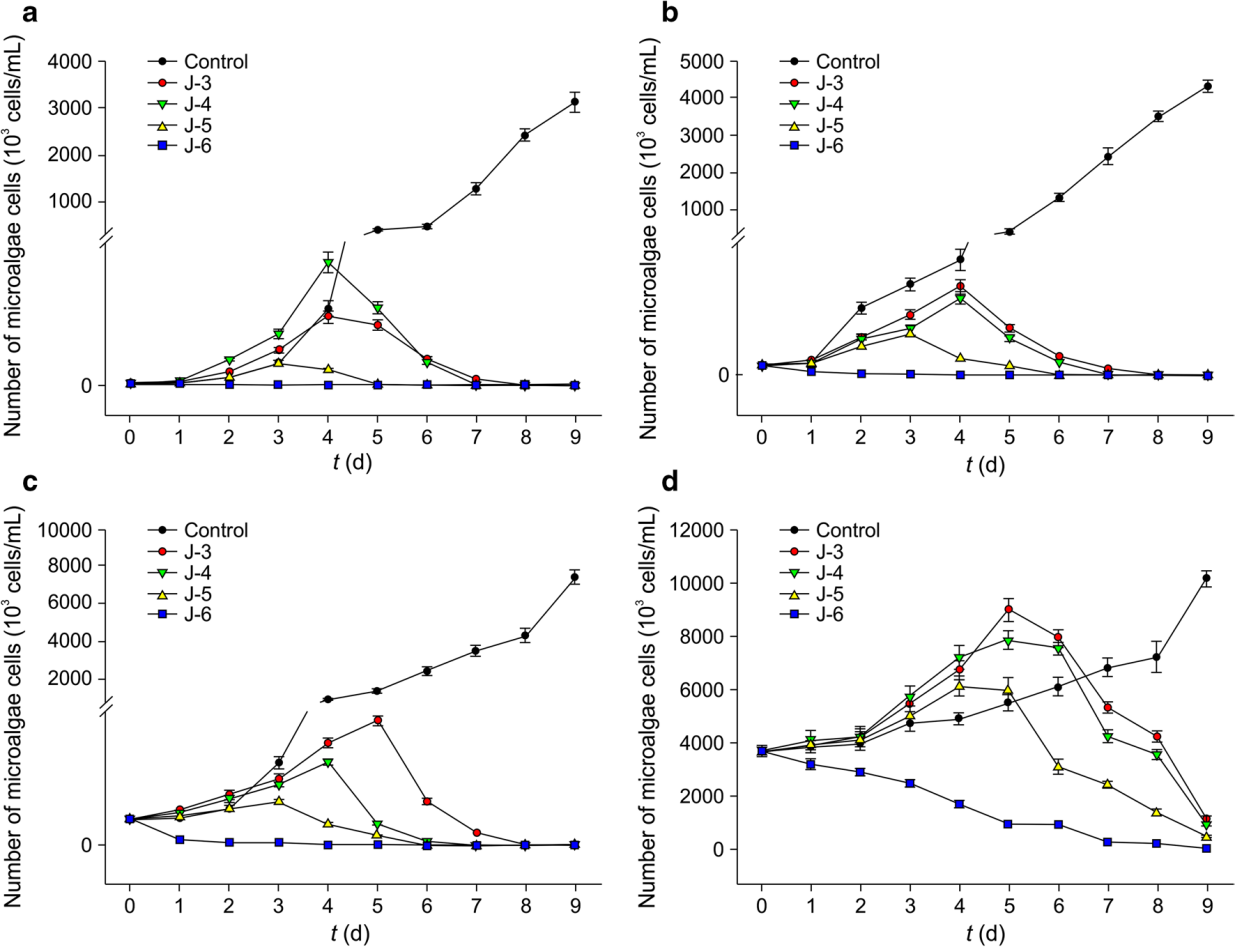
Alginolytic effects of different initial concentrations of CZBC1 on *Oscillatoria planctonica* at different initial concentrations. **a** 10^3^ cells/mL. **b** 10^4^ cells/mL. **c** 10^5^ cells/mL. **d** 10^6^ cells/mL

When the initial cell count of *O. planctonica* was 10^3^ cells/mL (Fig. 9a), cells in the control group exhibited logarithmic growth and cell count was 3.14 × 10^6^ cells/mL on day 9, which was significantly higher than that of the bacterial suspension group (*P* < 0.05). The cell count first increased before decreasing in the J-3, J-4, and J-5 groups, after the bacterial suspension was added, and peaked on day 4, day 4, and day 3, respectively, which was later than that observed for *O. chlorina* and *O. tenuis*. The cell count on these days was 1.30 × 10^5^ cells/mL, 1.97 × 10^5^ cells/mL, and 3.50 × 10^4^ cells/mL, respectively. Subsequently, the cell count of the three groups started to decrease and all algal cells had died by day 8, day 7, and day 6, respectively. In the J-6 group, algal cell count started to decrease after the bacterial suspension was added, and all algal cells had died by day 3.

When the initial cell count of *O. planctonica* was 10^4^ cells/mL (Fig. 9b), cells in the control group gradually increased as culture duration increased and cell count was 4.31 × 10^6^ cells/mL on day 9, which was significantly higher than that of the bacterial suspension group (*P* < 0.05). The cell count increased in the J-3, J-4, and J-5 groups after the bacterial suspension was added, reaching a maximum of 1.72 × 10^5^ cells/mL, 1.50 × 10^5^ cells/mL, and 8.13 × 10^4^ cells/mL, respectively. Subsequently, the *O. planctonica* cell count of the three groups started to decrease, and all algal cells had died by day 8, day 7, and day 6, respectively. In the J-6 group, algal cell count started to decrease after the bacterial suspension was added, and all algal cells had died by day 4.

When the initial cell count of *O. planctonica* was 10^5^ cells/mL (Fig. 9c), cells in the control group gradually increased as culture duration increased and cell count was 7.43 × 10^6^ cells/mL on day 9, which was significantly higher than that of the bacterial suspension group (*P* < 0.05). The cell count increased in the J-3, J-4, and J-5 groups after the bacterial suspension was added. In the J-3 group, cell count peaked on day 5 with a cell count of 6.44 × 10^5^ cells/mL, in the J-4 group it peaked on day 4 with a cell count of 4.29 × 10^5^ cells/mL, and in the J-5 group it peaked on day 3 with a cell count of 2.22 × 10^5^ cells/mL. Subsequently, the cell count of the three groups started to decrease, and all algal cells had died by days 8, 7, and 6, respectively. In the J-6 group, algal cell count started to decrease after the bacterial suspension was added. The alginolysis rate on day 1 was 83.71%, and all algal cells had died by day 5.

When the initial cell count of *O. planctonica* was 10^6^ cells/mL (Fig. 9d), cells in the control group gradually increased as culture duration increased and cell count was 1.01 × 10^7^ cells/mL on day 9. After adding the bacterial suspension, *O. planctonica* in the J-3, J-4, and J-5 groups first increased before decreasing, reaching a maximum of 8.96 × 10^6^ cells/mL, 7.82 × 10^6^ cells/mL, and 5.97 × 10^6^ cells/mL, respectively. The growth rate of *O. planctonica* cells in the first 5 days was faster in these three groups than in the control group. The cell counts of these three groups decreased, reaching 1.17 × 10^6^ cells/mL, 9.88 × 10^5^ cells/mL, and 5.3 × 10^5^ cells/mL, respectively, on day 9. In the J-6 group, algal cell count started to decrease after the bacterial suspension was added, and cell count on day 9 was 7.67 × 10^4^ cells/mL.

## Discussion

Algicidal bacteria can perform both direct and indirect alginolysis (Yang et al. 2013; Sun et al. 2016). Reports on direct alginolysis include myxobacteria that directly contact and lyse cyanobacteria such as *Anabaena, Microcystis, Aphanizomenon,* and *Oscillatoria* species, as well as other microalgae (Peterson et al. 1993); bacteria belonging to the genera *Cytophaga* and *Saprospira* that contact and specifically lyse dinoflagellates and diatoms (Furusawa et al. 2003); and a strain of *Pseudomonas putida* that kills *Stephanodiscus* sp. by direct alginolysis (Kang et al. 2005). While the majority of algicidal bacteria affect the growth of algae indirectly by excreting algicidal compounds or extracellular products (Demuez et al. 2015). Li et al. (2015) isolated the *Bacillus* strain LZH-5 from Lake Tai that exhibits strong alginolytic effects on *M. aeruginosa.* This bacterium secretes alginolytic compounds to indirectly lyse algae. Lee et al. (2000) isolated the *Pseudoaltermonas* strain A28, and found that its supernatant could kill *Skeletonema costatum*. After filtering the supernatant of A28 using a 10,000-Mw filter, these researchers found that the concentrated supernatant exhibited alginolytic activity, suggesting that A28 can synthesize extracellular macromolecules for alginolysis. In fact, A28 produces a serine protease responsible for alginolysis (Lee et al. 2000). In addition, some bacteria can lyse algal cells through the two methods, simultaneously. An example was reported by Pei et al. (2005) who found that algicidal bacteria cells and their sterile filtrate could both kill algal cells. In the present study, CZBC1 lysed *O. chlorina* and *O. tenuis* through direct alginolysis, while its extracellular products lysed *O. planctonica* through indirect alginolysis.

The alginolytic effects of algicidal bacteria on algae are associated with bacterial concentration. Su et al. (2007) and Huang et al. (2013) showed that a certain concentration is required for alginolysis. Within a certain range, the higher the concentration of algicidal bacteria, the higher the alginolytic rate, and the more significant the alginolytic effects. Kang et al. (2015) Suggested the threshold density (10^5^ cell/mL) of *Lactobacillus paraplantarum* might be a prerequisite for achieving successful termination of natural blooms. And Shao et al. (2014) reported that the algicidal effect of *Bacillus* sp. B50 occurred at least at 1.9 × 10^6^ cfu/mL, and no algicidal effect was observed at cell densities lower than 1.9 × 10^5^ cfu/mL. Chen et al. (2015) studied the characteristics of the algicidal bacterium *Pseudomonas aeruginosa* JM1 and found that its algicidal effect increased with increasing initial algal cell density and amount of fermentation fluid. Bacterial cells at 10^11^ cfu/mL could inhibit algal cells growth alone and could promote the algicidal effect of the fermentation fluid at 10^5^ cfu/mL and above. In the present study, we analyzed the effects of different initial CZBC1 concentrations on the growth of three *Oscillatoria* species. The results indicated that when the initial concentration of CZBC1 was 10^6^ cfu/mL, this strain exhibited alginolytic effects on *O. chlorina*, *O. tenuis*, and *O. planctonica* at initial concentrations of 10^3^–10^6^ cfu/mL. When the initial concentration of CZBC1 was lower (10^3^ cfu/mL), its inhibitory effects on the different initial concentrations of *O. chlorina*, *O. tenuis*, and *O. planctonica* were delayed by 2–5 days. However, the cell counts significantly decreased compared with those of the control group, showing significant alginolysis.

Most studies conducted so far have considered chlorophyll content to assess the alginolytic effects of algicidal bacteria (Su et al. 2007; Huang et al. 2013; Shao et al. 2015), although it is debated whether chlorophyll content can be used to assess alginolytic effects after lysis of algal cells by algicidal bacteria. In the present study, the algicidal strain CZBC1 showed direct alginolytic effects on *O. chlorina* and *O. tenuis* and indirect alginolytic effects on *O. planctonica*, and we directly determined algal cell count to more accurately assess alginolytic effects. Comparisons performed in the present study revealed that when the initial algal cell count was 10^3^–10^5^ cells/mL, the alginolytic effects of CZBC1 appeared earlier on *O. chlorina* and *O. tenuis* than on *O. planctonica*, and direct alginolysis was used to rapidly achieve alginolysis.

The cell count of *O. chlorina* in aquaculture ponds is usually 10^3^–10^6^ cells/mL (Liang et al. 2007; Liu et al. 2009), and this species is often one of the dominant cyanobacteria in prawn aquaculture ponds (Liu et al. 2011). It damages *Litopenaeus vannamei*, and the degree of damage is associated with cell count (Xu et al. 2017). When the *Oscillatoria* sp. cell count in water bodies reaches 10^6^ cells/mL, both live and dead algal cells can severely affect the survival of prawns. When *Oscillatoria* sp. cell count in water bodies decreases to values below 10^4^ cells/mL, prawn mortality rate greatly decreases (Cao 2014). According to the results of our study, *Oscillatoria* species can be controlled in aquaculture systems by regularly applying the algicidal strain CZBC1 (10^3^ cfu/mL) once every 7–10 days. After cyanobacteria blooms, CZBC1 application should be increase to 10^6^ cfu/mL 2–3 times to control *Oscillatoria* and other cyanobacteria.

In summary, our study examined the alginolytic effects of the algicidal strain CZBC1 on three *Oscillatoria* species and compared the alginolytic effects of different initial concentrations of CZBC1 on different cell counts of *O. chlorina*, *O. tenuis*, and *O. planctonica*. Our study showed that CZBC1 can directly lyse *O. chlorina* and *O. tenuis* and indirectly lyse *O. planctonica*. The higher the concentration of this algicidal strain, the more significant its alginolytic effects against the three *Oscillatoria* species. We also determined the effective application concentration of CZBC1. We will carry out further studies on the transformation routes of algal debris after cyanobacteria lysis by algicidal bacteria, and the residual form and attenuation effects of cyanobacterial toxins. At the same time, the physiological and molecular mechanisms of the alginolytic effects of CZBC1 will also be studied.

## Data Availability

The datasets supporting the conclusions of this article are included within the article.
